# Fine population structure analysis method for genomes of many

**DOI:** 10.1038/s41598-017-12319-1

**Published:** 2017-10-03

**Authors:** Xuedong Pan, Yi Wang, Emily H. M. Wong, Amalio Telenti, J. Craig Venter, Li Jin

**Affiliations:** 1 Ministry of Education Key Laboratory of Contemporary Anthropology, Collaborative Innovation Center for Genetics and Development, School of Life Sciences, Fudan University, Shanghai, China; 2 Human Longevity Inc., San Diego, CA 92121 USA; 3 State Key Laboratory of Genetic Engineering, Collaborative Innovation Center for Genetics and Development, School of Life Sciences, Fudan University, Shanghai, China

## Abstract

Fine population structure can be examined through the clustering of individuals into subpopulations. The clustering of individuals in large sequence datasets into subpopulations makes the calculation of subpopulation specific allele frequency possible, which may shed light on selection of candidate variants for rare diseases. However, as the magnitude of the data increases, computational burden becomes a challenge in fine population structure analysis. To address this issue, we propose fine population structure analysis (*FIPSA*), which is an individual-based non-parametric method for dissecting fine population structure. *FIPSA* maximizes the likelihood ratio of the contingency table of the allele counts multiplied by the group. We demonstrated that its speed and accuracy were superior to existing non-parametric methods when the simulated sample size was up to 5,000 individuals. When applied to real data, the method showed high resolution on the Human Genome Diversity Project (HGDP) East Asian dataset. *FIPSA* was independently validated on 11,257 human genomes. The group assignment given by *FIPSA* was 99.1% similar to those assigned based on supervised learning. Thus, *FIPSA* provides high resolution and is compatible with a real dataset of more than ten thousand individuals.

## Introduction

Analyses of genetic structure of extant populations shed light on the evolutionary history of our species and provide information on etiology of diseases under the interplay of genetic and environmental factors.^1–7^ For example, filtering by the highest allele frequency in any subpopulation of the ExAC dataset is a powerful approach in the selection of candidate protein-altering variants for rare diseases^8^. Recently, studies involving large number of genomes or exomes are emerging^6,8–10^. In fact, the number of whole-genome or whole-exome sequences in populations is expected to reach hundreds of thousands in the near future. Thus, a method for analyzing fine population structure with a large number of individuals is much needed.

Population structure analysis is a process of inferring individual ancestry from genotypic information^11^. Genetically similar individuals are grouped together, and the proportion of each individual’s ancestries can be estimated. In recent decades, the estimation of the contributing ancestries has received much attention, given prevailing admixture in human evolutionary history^12,13^. *Structure* is a representative method for inferring ancestry proportion. After the introduction of the probabilistic model of Pritchard, Stephens and Donnelly (PSD model)^14^, several methodological improvements have been made to improve the computational efficiency^15–19^. The most recent advance, TeraStructure^19^, is capable of handling 10^12^ observed genotypes.

Though the technical advances have enabled proportional ancestry analysis on datasets of millions of individuals,^19^ the resolution of the proportional ancestry models may not be as good as that of individual-based analyses, which has fewer parameters^14^.

Individual-based population structure analysis can be non-parametric or parametric. The most widely used^20–22^ non-parametric method is principal component analysis (PCA). Through dimension reduction, the top principal components (PCs), which explain the majority of the genetic variation among the individuals, are obtained. However, the interpretation of the PCs is not always straightforward^18^. On the other hand, an example of parametric methods, *fineSTRUCTURE* ^23^, which has high resolution^4,11^, is computationally intensive because of its O(${N}_{ind}^{2}$) complexity, where *N*_*ind*_ is the sample size of the data.

In an effort to overcome the computational burden of fine population structure analysis when the sample size is relatively large, we propose a new route to explore fine population structure, which is referred to as Fine Population Structure Analysis (*FIPSA*). *FIPSA* provides optimized speed and resolution by linkage disequilibrium (LD)-based pruning of single nucleotide variants (SNVs), and is compatible with a real dataset of more than ten thousand individuals.

## Methods

Fine Population Structure Analysis (*FIPSA*) attempts to determine the best individual assignment by maximizing the genetic differences among the subgroups. We test the performance of the absolute value of the allele frequency differences between sub groups (*DAF*), *Fst* and likelihood ratio (*LR*) on simulated datasets (details in Results and Discussion) and select *LR* as the statistic to describe genetic differences among the subgroups. We then illustrate how to use likelihood ratio (*LR*) to describe genetic differences among subgroups. Then we describe how to find the best partition for all individuals by maximizing the *LR*. Finally, we propose an ad-hoc approach so that the best subpopulation count (*K*) is chosen.

### Calculating LR

We first consider a situation of one polymorphic site with *n* alleles. If we want to classify all individuals to *K* groups based on the genotype of this loci, first, an allele count table of *n* by *K* must be made (Table 1). Likelihood ratio is calculated following equation (1).

1 $$LR=\sum _{i=1}^{n}\sum _{k=1}^{K}{O}_{ik}\,\mathrm{log}({O}_{ik}/{E}_{ik})$$

where

$$\,{E}_{ik}=\,\sum _{i=1}^{n}{O}_{ik}\ast \,\sum _{k=1}^{K}{O}_{ik}\underset{i=1}{\overset{n}{/\sum }}\sum _{k=1}^{K}{O}_{ik}$$

**Table 1:** Calculating LR for one loci.

	Allele 1 count	Allele 2 count	…	Allele n count
Group 1	*O*_11_	*O*_12_		*O*_1*n*_
Group 2	*O*_21_	*O*_22_		*O*_2*n*_
…				
Group K	*O*_*K*1_	*O*_*K*2_		*O*_*Kn*_

Table of an allele cross group for one loci, assuming *n* alleles for the loci and the grouping of individuals to *K* clusters.

The *LR* changes when the individual group assignment changes. By maximizing the *LR*, the best group assignment is determined.

For multiple loci that are mutually independent, let the loci count of a dataset be *S*. Then, the total *LR* of this dataset (denoted as *LR*_*u*_) can be calculated according to equation (2).

2 $$L{R}_{u}=\sum _{{\rm{s}}=1}^{S}\sum _{i=1}^{n}\sum _{k=1}^{K}{O}_{ik{\rm{s}}}\,\mathrm{log}({O}_{iks}/{E}_{iks}).$$

The existence of LD would violate the independence assumption of the *LR* calculation. Thus, pruning SNVs to reduce LD is suggested.

### Finding the best group assignment

Given the genotypes for a set of individuals, the *LR* corresponding to a certain state of individual assignment could be calculated. Let *X* represent the genotype, and *Z*_*best*_ represent the best individual assignment which can be estimated using equation (3).

3 $${Z}_{best}=argmax\{LR(X|Z)\}$$

The optimal solution of *Z* corresponds to the absolute maximum value of the *LR*, which can be solved using brute force methods. However, in practice, the computational burden of brute force methods becomes severe even for datasets with 30 individuals.

To improve computational efficiency, we implemented a simulated annealing based approach in order to search for the maxima of the *LR*. Considering a Markov chain with a stationary distribution $\pi (E)=k{e}^{(-\frac{E}{T})}$, and let $E=-LR(X|Z)$, as described by Kirkpatrick *et al*.^24^, the maxima of the *LR*(*X*|*Z*) is reached when the chain described in equation (4) converges to equilibrium.

4 $${x}_{i}\,\propto {e}^{(\frac{LR(X|{Z}_{i})}{{T}_{i}})}$$

For each *Z*_*i*_ ∈ *Z* in equation (4), the neighbors of *Z*_*i*_ (denoted as ${Z}_{i}^{^{\prime} }$) are a set of *K* elements, which differ from *Z*_*i*_ by the group assignment of a randomly chosen individual *r* (${z}_{ir}^{^{\prime} }=1,2,\ldots ,\,K$). The initial grouping state of the Markov chain (denoted as *Z*_0_) is generated by randomly assigning each individual to *K* subgroups. For any state *Z*_*i*_ on the Markov chain, let *z*_*ij*_ be the group assignment of individual *j*. We randomly choose one individual *r*, of which the group assignment is *z*_*ir*_. According to the property of the Boltzmann distribution, it is easy to show that the conditional distribution for individual *r*’s group assignment follows equation (5), where *n* is the total individual count and *i* is the index of discrete time.

5 $$p({z}_{ir}^{^{\prime} }|{z}_{i1},\ldots ,{z}_{i(r-1)},{z}_{i(r+1)},\,\ldots ,{z}_{in}\,)=\,{e}^{(-\frac{LR(X|{Z}_{i})}{{T}_{i}}+\frac{LR(X|{Z}_{i}^{^{\prime} })}{{T}_{i+1}})}$$

Thus, the probability of changing *z*_*ir*_ to ${z}_{ir}^{^{\prime} }$ follows equation (6), which defines the probability of jumping from the current state to its neighborhood state.

6 $$p({z}_{ir}^{^{\prime} }=C)=\frac{{e}^{(-\frac{LR(X|{Z}_{i})}{{T}_{i}}+\frac{LR(X|{z}_{ir}^{^{\prime} }=C)}{{T}_{i+1}})}}{{\sum }_{j=1}^{K}{e}^{(-\frac{LR(X|{Z}_{i})}{{T}_{i}}+\frac{LR(X|{z}_{ir}^{^{\prime} }=j)}{{T}_{i+1}})}},\,C\in \{1,2,\ldots ,K\}$$

Then the group assignment for each individual is updated using a hill-climbing process, during which the *LR* gradually increases. After a sufficient number of iterations, the maximum *LR* value and its corresponding grouping state *Z* is recorded, which represents the best individual assignment (*Z*_*best*_).

### Testing the existence of population structure

A non-homogeneous population can be partitioned into at least two subpopulations (*i.e. K* = 2). Thus, we here propose a permutation approach for *K* = 2 to test the existence of population structure. Assuming independence for each SNV, we randomly shuffle alleles among all of the individuals in order to break down existing population structure. Repeating this process numerous times, typically twenty, results in permutated datasets without population structure. *FIPSA* is then run for *K* = 2 on both the permutated datasets and the original dataset. If the *LR* for the original dataset is larger than the *LR* for the permutated datasets, it indicates that population structure exists (details in Supplementary Methods and Supplementary Discussion).

### Choice of K

If population structure exists, we then begin to resolve the structure. Many ideas have been proposed for the choice of *K*. As a representative non-parametric method, *Eigenstrat* ^20^ implemented a TW test in order to determine *K* while the classical model-based *Structure* ^14^ uses BIC (Bayesian information criteria) for the choice of *K*. Recently, Lawson *et al*.^23^ successfully incorporated *K* into the likelihood and chose *K* via the RJMCMC (reverse jump MCMC) technique. Despite the frequent attention given to the matter, the choice of *K* has been notoriously difficult. We strongly suggest setting the *K* using biological knowledge. At the same time, we propose an ad-hoc approach to select *K*. More discussion on choice of K can be found in Supplementary.

### Maximum informative K (*K*_*max*_*info*_)

We calculate the second derivative of the *LR* on *K* (*SOD*(*K*)) as

$$SOD(K)=LR(K)-(LR(K-1)+LR(K+1))/2$$

Based on *SOD*(*K*), we define *K*_*max*_*info*_ following equation (7). The characteristic of *K*_*max*_*info*_ is reflected by the sudden drop in the fluctuation of *SOD*(*K*) over *K*, and can be summarized by the following two criteria:

1. globally, *SOD*(*K*_*max*_*info*_) ≫ *SOD*(*K*), when *K* > *K*_*max*_*info*_
2. locally, *SOD*(*K*_*max*_*info*_) ≫ *SOD*(*K*_*max*_*info*_+1)

These two criteria are then combined in order to get the discriminant for *K*_*max*_*info*_.

7 $$\begin{array}{c}{K}_{{\rm{\max }}\_info}=argmax\{\frac{SOD{(K)}^{2}}{{\sum }_{i=K}^{{K}_{max}-1}SOD{(i)}^{2}}{(1-\frac{SOD(K+1)}{SOD(K)})}^{2}:K\in \{2,\ldots ,({K}_{max}-3)\}\},\\ \quad \quad \quad \quad {\rm{if}}\,SOD(K) > 0\,\& \& \,SOD(K) > SOD(K+1)\end{array}$$

## Results

### Choosing LR as the statistic to describe genetic differences among sub groups

To describe the genetic differences among subgroups, a straightforward way is to calculate the absolute value of the allele frequency differences between subpopulations, which we denote as *DAF* (delta allele frequency). This statistic was tested on simulated data containing two subpopulations, and it was shown to be informative (Supplementary Fig. 1, simulation details are in Supplementary Discussion). However, this statistic could not be directly extended to situations in which there were more than two subpopulations; the ‘workhorse’^5^ *Fst* does not have this drawback. In addition, *Fst* naturally measures the divergence of subpopulations. Thus, we further tested *Fst* ^25^ on the same simulated data. Unexpectedly, the performance of *Fst* was shown to be poor (Supplementary Fig. 1). Finally, inspired by the McDonald-Kreitman test, which assesses the significance of the likelihood ratio (*LR*) of a contingency table in which SNV counts classified by a functional annotation cross SNV counts classified by evolutionary history, we chose the *LR* to describe the genetic differences among subpopulations. On simulated data, maximizing the *LR* resulted in better performance than what was shown for maximizing the two other popular statistics (Supplementary Fig. 1), *Fst* and *DAF*. We also discussed other statistics in the Discussion section.

### Simulated data

For comparison, we tested the *ChromoPainter* unlinked model and *fineSTRUCTURE* (denoted as *FS-CPU*), *K-means* (cascadeKM function in R “vegan” package^26^) and *FIPSA* in parallel (details in Supplementary Results) on the same simulated datasets. We simulated datasets with 500 individuals^11^ and 5,000 individuals respectively, using the demographic model described in Supplementary Fig. 2 and Supplementary Results. Within each scenario, the simulated datasets comprised independent SNVs. By adding more independent SNVs into the simulated datasets, the classification accuracy of the three methods improved. We stopped adding SNVs when the performance of the methods plateaued. Finally, the number of SNVs in the simulated datasets ranged from 3,000 to 25,000. The performance of the three methods was measured by Adjusted Random Index (ARI), which was a value ranges from 0 to 1. A value of 1 means the inferred grouping is the same with the truth. A value of 0 means the inferred grouping is completely random compared with the truth.

### Scenario: 500 individuals

**Figure 1:**
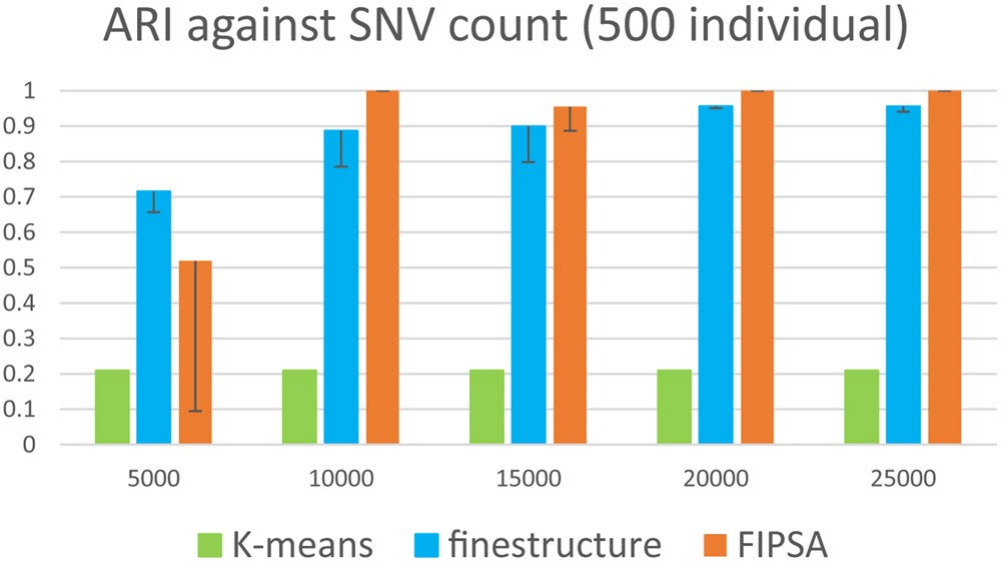
Three methods’ comparison on simulated dataset with 500 individuals. ARI (adjusted random index) against the SNV count, assuming *K* was unknown. Five replicates were used to calculate the standard deviation of ARI.

For each SNV count, five datasets were randomly generated in order to give the mean and standard deviation of accuracy (Fig. 1). The *K-means* method performed the worst in this situation. The low ARI of the *K-means* was a result of the choice of *K*. Although the Calinski criteria is shown to be the best criteria for *K-means* on simulated data, it repeatedly failed to make the right choice in the current scenario. *FineSTRUCTURE* was shown to be better than *FIPSA* when the information in the dataset was relatively insufficient (SNV count <10,000). Compared to the situation in which it was assumed *K* was known, the choice of *K* negatively affected the performance of *FIPSA*. However, when information in the dataset was relatively abundant (SNV count > = 10,000), *FIPSA* was shown to slightly outperform *fineSTRUCTURE*, indicating that the choice of *K* is relatively good when sufficient information is present.

### Scenario: 5,000 individuals

**Figure 2:**
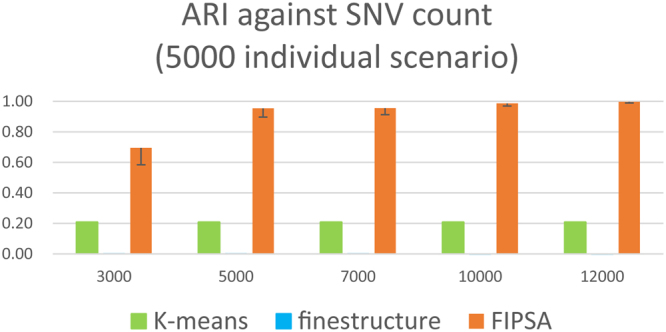
Three methods’ comparison on simulated dataset with 5,000 individuals. ARI (adjusted random index) against SNV count, assuming *K* was unknown. Five replicates were used to calculate the standard deviation of ARI.

The size of each subpopulation increased from 100 to 1,000, and we applied the same procedure. Even with a high number of burn-in iterations and sample iterations (*fineSTRUCTURE* -x 100000 –y 100000), *fineSTRUCTURE* failed in scenarios with a large sample sizes (Fig. 2). The chosen *K* for *fineSTRUCTURE* was always above one hundred.

**Figure 3:**
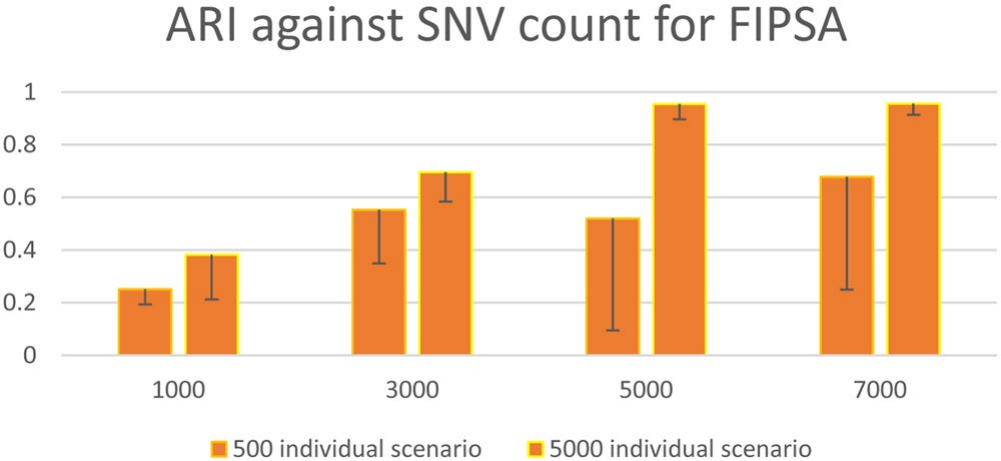
Comparison of *FIPSA*’s performance between 500 individual scenario and 5,000 individual scenario. ARI (adjusted random index) against the SNV count, the 500 individual scenario compared with the 5,000 individual scenario.

In this situation, assuming *K* was unknown, the performance of *FIPSA* was even better than what was shown for a small sample size, indicating that *FIPSA* favors scenarios with a large sample size (Fig. 3).

According to the definition of the likelihood ratio for *FIPSA* (equation (2)), it is evident that under certain degrees of freedom, the larger the sample size is, the bigger the delta of the *LR* of the different group assignments is. Thus, *FIPSA* gains more power as the sample size increases. Also, there is a larger standard deviation in the accuracy in the 500 individual scenario as compared with the 5,000 individual scenario, indicating that the choice of *K* performs better in a ten-fold sample size scenario.

In conclusion, when the sample size reaches 5,000, *FIPSA* performed the best on unlinked data among the three methods.

### Speed evaluation

**Figure 4:**
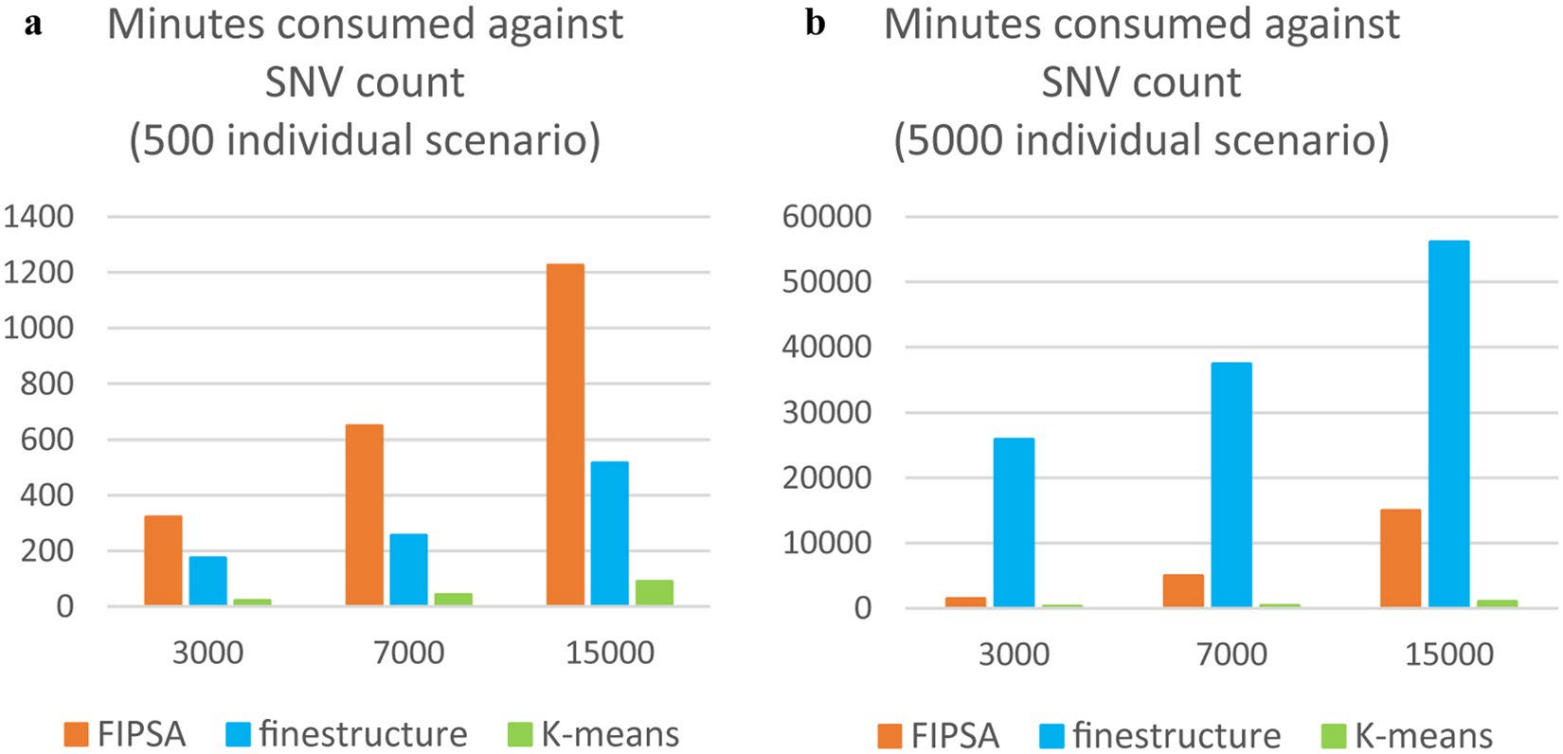
Time consumption against the SNV count for the three methods. (**a**) 500 individual scenario; (**b**) 5,000 individual scenario. Details of parameters are in Supplementary Results.

We compared runtime performance of *K-means*, *fineSTRUCTURE* and *FIPSA* (details in Supplementary Results) for the 500- and 5,000-individual simulated datasets, respectively (Fig. 4). Runtime (in minutes) is reported based on performance on a single thread.

Figure 4 shows that the *K-means* is fast. However, the *K-means* shows poor accuracy in both scenarios (Figs 1 and 2), mostly as a result in the choice of *K*. The inverse performance between *fineSTRUCTURE* and *FIPSA* on the different sample sizes indicates that the two methods favor different parametric spaces in terms of speed. Let *N*_*ind*_ be the individual count and *N*_*SNP*_ be the SNV count of a dataset. Comparing Fig. 4a with Fig. 4b, the runtime of *fineSTRUCTURE* increased by about 100 times as the sample size increased by 10 times, consistent with its O(${N}_{ind}^{2}$) complexity. However, its complexity is not dependent on *N*_*SNP*_; the SNV count only affected the speed of *choromopainter*, rather than *fineSTRUCTURE*. On the contrary, the runtime of *FIPSA* was approximately proportional to *N*_*ind*_ and *N*_*SNP*_ both, rendering its relative speed advantage in the huge sample size scenario over the pairwise distance based methods. As a result, *fineSTRUCTURE* is faster than *FIPSA* in scenarios in which there is only a moderate sample size (several hundred individuals), and slower in scenarios in which there is a large sample size (several thousand individuals). However, the results of the evaluation of speed may be very different under other simulation parameters rather than the ones used here. For example, *FIPSA* needs more iterations to converge when *K* is big (data not shown).

In conclusion, *FIPSA* showed an advantage in speed compared to *fineSTRUCTURE* when the sample size was 5,000, and the *K* was 5. The difference is expected to be increasingly significant when sample size is increased further.

### Real data

The HGDP^27^ East Asian and European datasets were used as representative datasets with known fine population structure for testing. As an independent validation, Human Longevity Inc. tested *FIPSA* on a large whole-genome sequencing dataset with 11,257 human genomes.

### HGDP East Asian dataset

**Figure 5:**
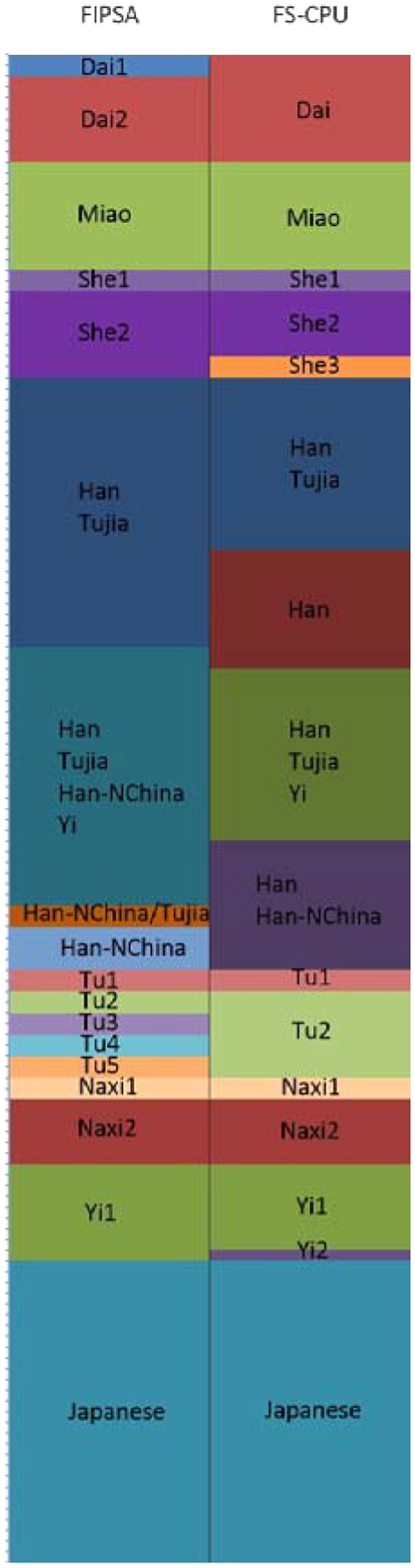
Comparison of *fineSTRUCTURE* (FS-CPU) and *FIPSA*’s clustering result on the HGDP East Asia dataset. Left column is *fineSTRUCTURE* with the *ChromoPainter* unlinked version (*FS-CPU*) result, with *K* = 16; Right column is *FIPSA*’s result, with *K*_max_*info*_ = 18. Calibration on the y axis corresponds to the individual count.

Lawson *et al*. has systematically evaluated the performance of the current representative individual-based methods on the HGDP East Asian dataset^11^, the population structure of which is fine-scale. On this dataset, after phasing and imputing the 140 whole-genome SNV array data using *shapeit2* ^28,29^, we compared the performance of the *choromopainter* unlinked version plus *fineSTRUCTURE* (*FS-CPU*) with *FIPSA* in Fig. 5.

The general classification of Dai, Miao, She, Tu, Naxi, Yi and Japanese were consistent between *fineSTRUCTURE*-*ChromoPainter* unlinked (*FS-CPU*) and *FIPSA*, with the only minor difference being that *FIPSA* showed finer population structure for Dai and Tu, while *FS-CPU* showed a clearer picture of She and Yi. Both methods separated the same Yi individual from the other Yi individual into a Han/Tujia or Han/Tujia/Han-NChina cluster. The clustering of Han, Tujia and Han-NChina was different between the two methods, although both methods grouped these individuals into four clusters. The results of *FS-CPU* were clearer than the results of *FIPSA* for Tujia and Han-NChina, where both populations were grouped into fewer clusters than when using *FIPSA*. *FIPSA* grouped the Han individuals into only two clusters; in contrast, *FS-CPU* grouped them into multiple clusters. The level of consistency between the two methods depended on the degree of differentiation between the populations. For a relatively strong signal, like the separation of Japanese from Chinese populations, both methods gave comparable results. For moderately strong signals, like the clustering of Dai, Miao, She, Naxi, Yi, Tu, both methods gave the same clustering boundary for each population, though the classifications within the populations had minor differences. For the more subtle structure of Han, Tujia and Han-NChina, the clustering results were less consistent.

### HGDP European dataset

The same approach was applied to the HGDP European dataset, which contained 157 individuals from 8 populations. The clustering results of *FS-CPU* and *FIPSA* is shown in Supplementary Fig. 3. *FS-CPU* was able to separate French from Italian individuals, while *FIPSA* was able to identify a unique Tuscan group.

### The deep sequencing of 10,000 human genomes

Human Longevity Inc. independently validated *FIPSA*’s performance on 162,997 SNVs’ genotypes from 11,257 individuals using *ADMIXTURE* as described by Telenti *et al*.^10^. Twenty replicates on *K* = 6 were performed. Each replicate was run on a single thread, and they took 24 to 40 hours. The best group assignment given by *FIPSA* had a 99.1% concordance with the assignment based on supervised learning: European (EUR), African (AFR), Central-South Asian (CSA), East Asian (EAS), Native American (AMR) and Middle Eastern (MDE). The 2,385 admixed individuals (ADMIX) identified by *ADMIXTURE* ^16^ were not considered in calculating the concordance (Table 2). The columns of Table 2 are group assignments based on supervised learning using *ADMIXTURE*. The rows of Table 2 are group assignments given by *FIPSA*.

**Table 2:** FIPSA’s individual assignment on 11,257 deeply sequenced human genomes.

	ADMIX	AFR	AMR	CSA	EAS	EUR	MDE
FIPSA Group 1 (EAS)	22	0	0	0	236	0	0
FIPSA Group 2 (AFR)	385	1234	0	0	0	0	0
FIPSA Group 3 (CSA)	104	0	0	280	0	0	0
FIPSA Group 4 (MDE)	777	0	0	0	0	77	265
FIPSA Group 5 (AMR)	435	0	132	0	0	0	0
FIPSA Group 6 (EUR)	662	0	0	0	0	6648	0

*FIPSA*’s individual assignment for *K* = 6 on 162,997 SNVs from 11,257 individuals (rows) and the group assignment based on super-populations described by the 1000 Genomes Project and Human Genome Diversity Project (column). AFR: African; AMR: American; CSA: Central-South Asian; EAS: East Asian; EUR: European; MDE: Middle Eastern.

In conclusion, *FIPSA* provided high resolution on the HGDP East Asian and European datasets and was computationally efficient even for a dataset with 11,257 individuals.

## Discussion

*FIPSA* was designed to group individuals by maximizing the genetic differences among subpopulations. We used the likelihood ratio as the statistic to measure genetic difference, and we implemented a simulated annealing based algorithm to determine the best group assignment. On a representative simulated fine population structure scenario proposed by Lawson *et al*.^11^, *FIPSA* showed considerable power in detecting the true structure as compared to existing non-parametric methods. Specifically, the resolution of *FIPSA* increased as sample size increased, out-performing representative non-parametric methods on simulated data. The runtime was better than other non-parametric methods when the sample size was in the thousands. Unlike pairwise-distance based methods, in which the complexity is the square of the sample size, the performance of *FIPSA* is linearly proportional to the sample size. Therefore, it is particularly suited for large datasets. *FIPSA* is also easy to use. Few parameters are needed, except for the restart time, which can be easily tuned. *FIPSA* tolerates missing values. It is also worth noting that theoretically, the number of allele per loci is flexible for *FIPSA*. Thus, in the future, it is possible for *FIPSA* to work on multi-allelic polymorphisms such as microsatellite data, which is widely used in forensic science.

Pritchard *et al*.^14^ proposed the PSD model, which has had numerous successes in the past 15 years^5,30,31^. Using the same model, Lawson *et al*. developed *fineSTRUCTURE* ^23^, which has a higher resolution and relatively good runtime performance. However, the computational burden may still be a concern for large datasets. In order to reduce the computational burden for fine population structure analysis in large datasets, we tried to describe population structure in a simpler perspective.

We used *LR* to describe population structure, which capture the degree of dependency of allele counts classified by the allele type on the allele counts classified by the population subgroup. The stronger the population structure is, the higher the dependency of those two classifications is shown to be. However, the *LR* may not always be the perfect choice because it may have less power when working on scenarios with extremely imbalanced subpopulation sample sizes. Also, there could be other options, such as choosing the group assignment with the most significant p-value in Fisher’s exact test or the mean number of pairwise differences between populations^32^. However, the excessive computational burden of the alternative methods on big data makes these attempts unfeasible.

To improve speed, we applied LD-based SNV pruning before running *FIPSA*, which implies that it may not perform as well as other methods that do take advantage of the LD information, such as *FS-CPL* (*ChromoPainter* linked and *fineSTRUCTURE*).

For relatively small datasets, existing methods may perform better than *FIPSA*; the purpose of proposing this method was to find a possible solution to explore fine population structure in large datasets. On HGDP East Asian dataset, *FIPSA* had relatively good resolution and was comparable to *FS-CPU*. At the same time, *FIPSA* was efficient on a real dataset with 162,997 genotypes from 11,257 individuals. *FIPSA* should also be able to work on datasets with 100K individuals at 100K SNVs. However, *FIPSA* does not take consideration of ancestral proportion. Thus, it is less useful for admixture analysis. Also, *FIPSA* compromises resolution for speed, by LD based SNV pruning.

The *FIPSA* software is available at: https://github.com/gelu0/FIPSA.git.

## Electronic supplementary material

Supplementary Materials
