# Supplementary material for: Fine population structure analysis method for genomes of many
Source: Sci Rep. 2017 Oct 3;7:12608. doi: 10.1038/s41598-017-12319-1 (PMC5626719; doi:10.1038/s41598-017-12319-1)
Supplement: Supplementary file 1 — Supplementary Materials [file 41598_2017_12319_MOESM1_ESM.pdf]

Fine population structure analysis method for genomes of many

Xuedong Pan[1,§], Yi Wang[1,§], Emily H. M. Wong[2], Amalio Telenti[2], J. Craig Venter[2], Li Jin[1,*]

[1] Ministry of Education Key Laboratory of Contemporary Anthropology and State Key Laboratory of Genetic Engineering, School of Life Sciences, Fudan University, Shanghai, China; [2] Human Longevity Inc., San Diego, CA 92121, USA.

Correspondence and requests for materials should be addressed to L.J. (email: lijin@fudan.edu.cn)

Supplementary Materials

Methods

Testing existence of population structure

We used the same demographical model proposed by Lawson *et al.*[1] to assess the fine population structure scenario. Three populations (A, B, C) were set to split simultaneously 3000 years ago, followed by a split from C to C1 and C2 2000 years ago, and a split from B to B1 and B2 1000 years ago (Supplementary Fig. 2). Linkage disequilibrium was not considered in the simulations. The procedure was as follows.

Step 1: independently simulate tens of thousands of segment using *SFS_CODE*[2]: *SFS_CODE* 5 1 -Td 0 0.3133 -TS 0.087084 0 1 -TS 0.087084 0 2 -TS 0.094777 1 3 -TS 0.102469 2 4 -TE 0.110162 -Tg 0 26.861714 -N 5000 -n 100 -A -L 1 2000

Step 2: randomly draw a single SNV from each segment to generate a dataset of certain counts of independent SNV.

Step 3: For each SNV count, step 2 is repeated 20 times to generate 20 simulated datasets.

Step 4: For each simulated dataset, 20 permutated datasets are generated to calculate pseudo p-value.

Results

Simulation details of demographic model of Supplementary Fig. 2

With a demographical model where there were three starting populations, we simulated unlinked SNVs using *SFS_CODE* for testing performance. The procedure was as follows.

Step 1: independently simulate tens of thousands of segment: *SFS_CODE* 5 1 -Td 0 0.3133 -TS 0.087084 0 1 -TS 0.087084 0 2 -TS 0.094777 1 3 -TS 0.102469 2 4 -TE 0.110162 -Tg 0 26.861714 -N 5000 -n 100 -A -L 1 2000.

Step 2: randomly draw a single SNV from each segment to generate a dataset of certain counts of independent SNV.

Step 3: For each SNV count, step 2 is repeated 5 times to generate 5 simulated datasets.

Details of the three methods evaluated on simulation data

ChromoPainter unlinked model and *fineSTRUCTURE*[3], *K-means*[4] were compared with *FIPSA*. *ChromoPainter* and *fineSTRUCTURE* are frameworks for dissecting fine population structure comprised of two steps. The first step is to construct a pairwise individual co-ancestry matrix using *ChromoPainter*, and the second step is the clustering of individuals based on the co-ancestry matrix using *fineSTRUCTURE*. *ChromoPainter* has two versions, the linked version and the unlinked version. Although the unlinked version is not as sensitive as the linked version, which gains a substantial advantage by modeling linkage disequilibrium following Li. *et al.*[5], the unlinked version is more representative because of its close relationship with classical methods such as Principal Component Analysis[6], *structure*[7] and *ADMIXTURE*[8]. Besides, the unlinked version of *fineSTRUCTURE* is non-parametric and assumes independence among the SNVs; thus, it is more comparable with *FIPSA*. We chose *K-means* as a representative of classical clustering methods because it is parameter-free and assumes loci independence as well. The Adjusted Random Index (ARI) was used to measure clustering accuracy for the three methods. The choice of K for the *K-means* is Calinski criteria, $K_{\max\_info}$ for *FIPSA* and not fixing K (default) for *fineSTRUCTURE*.

Parameters for speed evaluation

The parameter set for *K-means*: given K from 2 to 10, with each K having 100 iterations using the cascadeKM function in the vegan library, the Calinski criteria for K selection; *choromopainter unlinked and fineSTRUCTURE*: 100,000 burn-in iterations and 100,000 sample iterations for *fineSTRUCTURE*; *FIPSA*: given *K* from 2 to 10, each *K* with 100 iterations per individual, 10 replicates for each *K*, maximum informative *K* for choice of *K*. The evaluation of the speed was run on a desktop computer with Intel i7 3770 and 32 GB RAM.

Discussion

Simulated data containing two subpopulations

We used the ms[9] software to simulate a simple fine population structure scenario to compare *DAF*, *Fst* and *LR*. We simulated two populations with a sample size 50:50, split 1000 years, 2000 years and 5000 years ago, respectively. The parameters we considered were: Ne = 5000; Migration rate = 0.0312, which is equivalent to *Fst* = 0.0016 by migration drift equilibrium; 200 genes, each gene 40 kb; 100 replicates for each split year. For example, the 5000 years split scenario, the ms command is as follows:

```
ms 200 20000 -t 20 -r 200 2000000 -I 2 100 100 0.0312 -ej 0.01 2 1
```

Testing the existence of population structure

Using the demographical model proposed by Lawson *et al.*[1] as a representative case (Supplementary Fig. 2), we found that the permutation approach had much more power to detect population structure even when the simulated dataset only contained 1000 loci from 500 individuals. The LR for the permutated datasets was $2{,}369 \pm 31$ (twenty replicates), while the LR for the original dataset was $5{,}638$, which was much larger. The method had increased power as the SNV count, or the individual count, increased. Thus, for real datasets with hundreds of thousands of loci or thousands of individuals, this gain in power would be even larger.

However, this approach would result in some false positive results when used on real datasets. As linkage among loci is unavoidable in real situations, the permutation process would break down the linkage disequilibrium as well as the population structure, thus making a pseudo p value more significant for the linked dataset. Using chromosome 22 of the HGDP[10] East Asian dataset, which contains 9,660 SNVs from 140 individuals, as a representative real data of fine population structure, the permutation approach also proved to have more power, whereas the LR for the permutated datasets was $10{,}663 \pm 46$ (twenty replicates), while the LR for the original dataset was $17{,}258$.

Choice of K and K_{max_struct}

There is no perfect way to choose K. One possible solution is to use K_{max_info}. The K_{max_info} criteria is based on the likelihood ratio (LR) of each K. Thus, there is a chance that the LR for a certain K does not reach the maximum, which will lead to an inappropriate choice of K_{max_info}. To reduce this chance, the restart time for FIPSA should not be too small. Practically, for the HGDP East Asian dataset, the restart time was set to 60. Still, we did not exclude the possibility of an inappropriate K_{max_info}. Even so, the K_{max_info} criteria gives a suggestive rank for all of the calculated K, and the top ranked K is more likely to be better than the rest. Another drawback of using K_{max_info} is the setting of K_{max}. The K_{max} could not be set too large for a huge dataset, primarily because of the computational burden.

Besides K_{max_info}, we define K_{max_struct} (K with strongest structure) as the K in which the second derivative of the LR on K (SOD(K)) reaches the maximum.

$$SOD(K) = LR(K) - ((LR(K-1) + LR(K+1))/2$$

$$K_{max\_struct} = \text{argmax(SOD(K))}$$

The definition of K_{max_struct} was inspired by simulated data. When we ran *FIPSA* on simulated data with no population structure, we found LR to be approximately proportional to K. On the contrary, when we ran *FIPSA* on HGDP data, LR was not proportional to K, which was due to the presence of population structure. Thus, the K which reflects the strongest population structure (K_{max_struct}) is determined by second order difference of the LR over K (denoted as $SOD(K)$). And $SOD(K)$ is chosen as the statistic to assess K_{max_struct}. For HGDP dataset, the largest $SOD(K)$ corresponded with $K_{max\_struct} = 3$ (Supplementary Fig. 4).

The choice of K is based on heuristics. We strongly recommend that users specify K based on biological knowledge.

References

1 Lawson, D. J. & Falush, D. Population identification using genetic data. *Annu. Rev. Genomics Hum. Genet.* **13**, 337-361 (2012).

2 Hernandez, R. D. A flexible forward simulator for populations subject to selection and demography. *Bioinformatics* **24**, 2786-2787 (2008).

3 Lawson, D. J., Hellenthal, G., Myers, S. & Falush, D. Inference of population structure using dense haplotype data. *PLoS Genet.* **8**, e1002453 (2012).

4 Oksanen, J. *et al.* vegan: Community Ecology Package. https://cran.r-project.org (2016).

5 Li, N. & Stephens, M. Modeling linkage disequilibrium and identifying recombination hotspots using single-nucleotide polymorphism data. *Genetics* **165**, 2213-2233 (2003).

6 Price, A. L. *et al.* Principal components analysis corrects for stratification in genome-wide association studies. *Nat. Genet.* **38**, 904-909 (2006).

7 Pritchard, J. K., Stephens, M. & Donnelly, P. Inference of population structure using multilocus genotype data. *Genetics* **155**, 945-959 (2000).

8 Alexander, D. H., Novembre, J. & Lange, K. Fast model-based estimation of ancestry in unrelated individuals. *Genome Res.* **19**, 1655-1664 (2009).

9 Hudson, R. R. Generating samples under a Wright-Fisher neutral model of genetic variation. *Bioinformatics* **18**, 337-338 (2002).

10 Li, J. Z. *et al.* Worldwide human relationships inferred from genome-wide patterns of variation. *Science* **319**, 1100-1104 (2008).

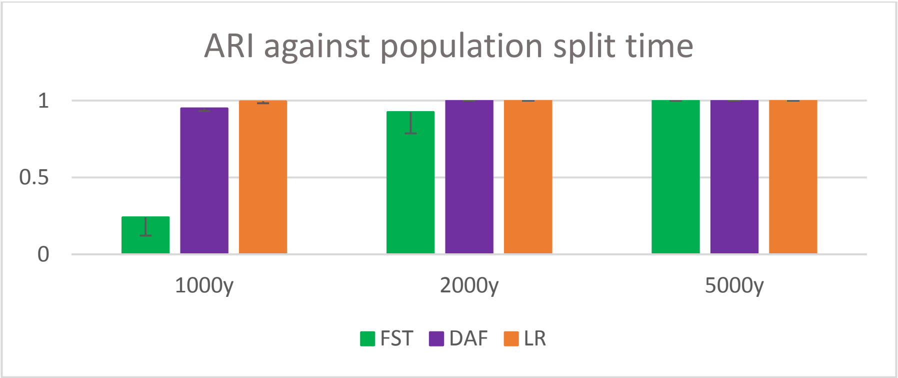

Supplementary Figure 1. Test three statistics' performance on simulated simplest fine population structure scenario. Three statistics' ARI (adjusted random index) against population split time.

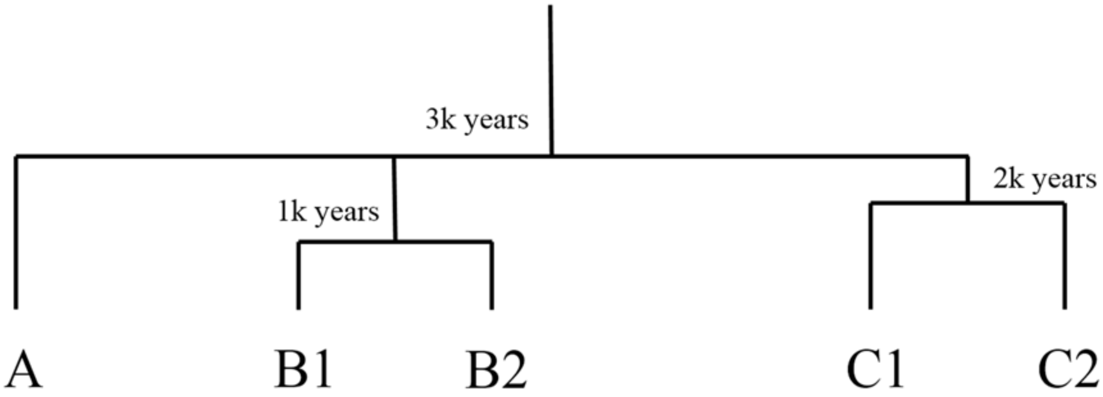

Supplementary Figure 2. Simulated fine population structure scenario. First, populations A, B, and C split simultaneously 3,000 years ago. Population C then splits into C1 and C2 2,000 years ago, and population B splits into B1 and B2 1,000 years ago.[1]

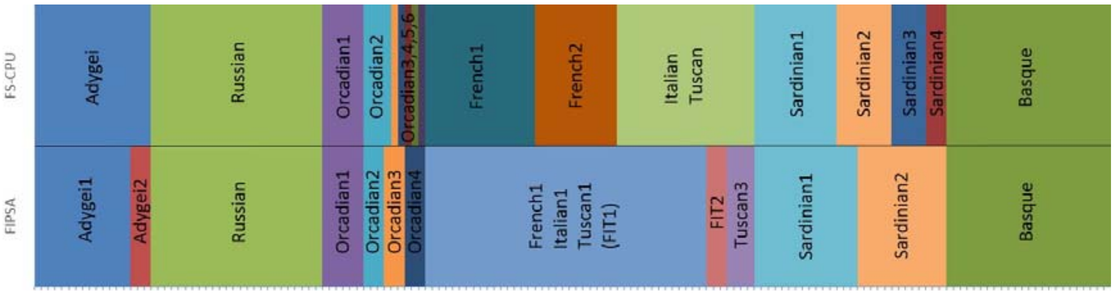

Supplementary Figure 3. Comparison of *fineSTRUCTURE* (FS-CPU) and *FIPSA*'s clustering result on the HGDP European dataset. Upper row is *fineSTRUCTURE* with *ChromoPainter* unlinked version's (FS-CPU) result, with K = 18; lower row is *FIPSA*'s result, with $K_{\max\_info}$ = 12. Calibration on x axis corresponds to individual count. FIT is short for French, Italian and Tuscan.

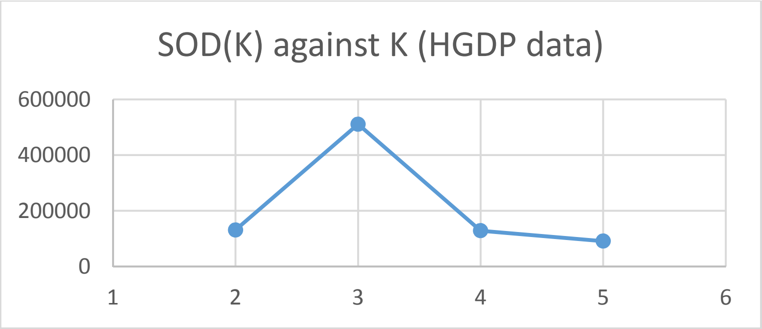

Supplementary Figure 4. Second derivative of *LR* over *K* (*SOD*(*K*)) against *K* for HGDP dataset. The y axis is *SOD*(*K*). The x axis is *K* (number of subpopulations).

References

1 Lawson, D. J. & Falush, D. Population identification using genetic data. *Annu. Rev. Genomics Hum. Genet.* **13**, 337-361 (2012).
